# Association between the age at onset of overweight and obesity and the subsequent risk of hypertension in Chinese adults

**DOI:** 10.1186/s12872-023-03347-z

**Published:** 2023-06-30

**Authors:** Hui Fan, Xingyu Zhang

**Affiliations:** 1 Department of Epidemiology and Health Statistics, School of Public Health, North Sichuan Medical College, Nanchong, Sichuan China; 2 Thomas E.Starzl Transplantation Institute, University of Pittsburgh Medical Center, Pittsburgh, USA

**Keywords:** Overweight, Obesity, Hypertension, Onset

## Abstract

**Background:**

Data on the impact of age at onset of overweight/obesity on the risk of hypertension are limited. We aimed to investigate the above-mentioned association in Chinese population.

**Methods:**

6700 adults who participated in at least three survey waves and were free of overweight/obesity and hypertension on first survey were included using China Health and Nutrition Survey. The age of participants at the onset of overweight/obesity (body mass index ≥ 24 kg/m^2^) and subsequent hypertension occurrence (blood pressure ≥ 140/90 mmHg or use of antihypertensive medication) were identified. We used the covariate-adjusted Poisson model with robust standard error to calculate the relative risk (RR) and 95% confidence interval (95%CI) to examine the relationship between the age at onset of overweight/obesity and hypertension.

**Results:**

There were 2,284 new-onset overweight/obesity cases and 2,268 incident cases of hypertension during an average 13.8-year follow-up period. Compared with the population without overweight/obesity, the RR (95% CI) of hypertension was 1.45 (1.28–1.65), 1.35 (1.21–1.52) and 1.16 (1.06–1.28) for overweight/obesity onset in participants aged < 38 years, 38–47 years, and ≥ 47 years, respectively. The risk of hypertension increased linearly with a decrease in age at onset of overweight/obesity (*P* < 0.001 for trend). The sensitivity analyses results were similar after excluding the participants taking antihypertensive medications or those with new-onset obesity or using waist circumference to define overweight/obesity.

**Conclusions:**

Our results emphasize the importance of assessing age at onset of overweight/obesity to prevent hypertension.

**Supplementary Information:**

The online version contains supplementary material available at 10.1186/s12872-023-03347-z.

## Introduction

Hypertension is a public health concern that has a high prevalence worldwide [1]. Hypertension contributes to cardiovascular disease and premature death [2]. Thus, early and accurate identification of individuals who are at risk of hypertension is essential to reduce the burden of hypertension and associated cardiovascular disease.

It is widely accepted that the conditions of overweight and obesity are major modifiable risk factors for hypertension [3]. Epidemiologic studies have confirmed that overweight and obesity increase the risk of hypertension [4]. Certain mechanisms, such as increased sympathetic nervous system activity and renin–angiotensin–aldosterone system activity, insulin resistance and pro-inflammatory cytokine release, are proposed to explain obesity-related hypertension [3]. Recently, the age of subjects at the onset of overweight and obesity has received substantial medical attention. Overweight and obesity onset during different periods of life may confer various health risks [5]. Additionally, the age at onset can be considered as a crude marker for the duration of obesity, given that adults with overweight and obesity rarely return to normalcy in terms of their weight [5–7].

The prevalence of obesity increased from 18.0% in 2007 to 21.2% in 2017 among Chinese adults aged 20–29 years [8]. Public health implication on the increasing trend in the prevalence and current high prevalence of obesity in young adults needed to be clarified. Kailuan cohort study used data form one China’s region and showed that the early onset of overweight was associated with higher risk of hypertension [9]. However, it is unclear regarding the impact of early onset of overweight and obesity on the risk of hypertension across China. To address these knowledge gaps, we assessed the association between the age at onset of overweight and obesity and hypertension using the China Health and Nutrition Survey (CHNS).

## Methods

### Study population

The CHNS is a continuous, nationwide, open cohort study [10]. It was designed to assess the impact of social and economic transformation in China on the health status of the population. The first survey wave was launched in 1989, and subsequent surveys were conducted at 2- or 4-year intervals. Data on a total of 10 survey waves through 2015 are available. The study used a multistage, random-cluster method to capture the sample. The participants were asked to complete a questionnaire and to undergo physical examinations during each of the survey waves. The details of the survey design, methods, and procedures have been described elsewhere [10]. All methods were carried out in accordance with the declaration of Helsinki. This study was approved by the institutional review boards of the University of North Carolina at Chapel Hill and the Institute of Nutrition and Health, Chinese Center for Disease Control and Prevention. All participants provided written informed consent before participating in the CHNS.

A total of 10,903 adult participants (≥ 18 years of age) who had complete and accurate data on sex, height, weight, and blood pressure (BP); who were free of disease (diabetes, myocardial infarction, apoplexy, bone fracture, asthma, and cancer) and pregnancy; and who agreed to complete at least three survey waves were eligible to participate in the current study. To assess the association of new-onset overweight and obesity with the subsequent incidence of hypertension, 3,346 participants who had overweight and obesity or hypertension at the time of the first survey wave were excluded. Moreover, 857 participants with an age at onset of overweight and obesity that was equal to or greater than the age at hypertension onset were also excluded. The final cohort comprised 6,700 adult participants. We identified four noteworthy observations in the final cohort, as follows: (1) a total of 6,700 adult participants were not classified as overweight or obese and did not have hypertension at the first survey wave; (2) the survey wave at which the participants had new-onset hypertension was considered as the final survey wave; (3) if participants did not have new-onset hypertension across any survey wave, the most recent survey wave was considered as the final survey wave; and (4) the age at onset of overweight and obesity was less than the age at onset of hypertension if participants had both new-onset overweight/obesity and hypertension (Figure S1).

### Primary exposure

Portable stadiometers were used by trained examiners to measure the participants’ height without shoes, and calibrated beam scales were used to measure body weight in light clothing. Body mass index (BMI) was calculated as body weight in kilograms divided by height in meters squared. Overweight and obesity were defined as a BMI of ≥ 24 kg/m^2^ [11]. We identified the age at onset of overweight and obesity. The tertiles of onset age were calculated. The upper and lower tertile cut-offs were 47 and 38 years, respectively. Consequently, participants were classified into four groups based on the age at onset of overweight/obesity. The four groups were those comprising the non-overweight/non-obese population and those with onset ages of < 38 years, 38–47 years and ≥ 47 years.

### Outcome assessment

Standard mercury sphygmomanometers were used by experienced physicians to measure BP three times on the right arm. The average of the three measurements was calculated and used for analysis. Hypertension was defined as a systolic BP (SBP)/diastolic BP (DBP) of ≥ 140/90 mmHg or self-reported use of antihypertensive medications [12].

### Covariates

We used structured questionnaires to collect data about sex (male/female), age, Han ethnicity (yes/no), urban residence (yes/no), completion of upper middle school education and above (yes/no), marital status (never/ married/ divorced, separated or widowed), alcohol intake (yes/no), smoking (never/current/past) and household asset score [13]. “Have you ever smoked cigarettes (including hand-rolled or device-rolled)?” and “Do you still smoke cigarettes now?” were used in the structured questionnaires to define smoking status. “Last year, did you drink beer or any other alcoholic beverage?” was used to define alcohol intake status. The household asset score was calculated based on the number of assets, each of which (color TV, refrigerator, microwave oven, electrical cooking pot, air conditioner, electric fan, and camera) was worth 1 point [13]. The length of follow-up was calculated as the number of years between the final and first survey years. Fat intake was assessed from three consecutive 24-hour dietary recalls and household food inventories using a Chinese food composition Table  [13]. Leisure physical activity was evaluated as the average number of hours per week spent engaged in the Martial arts, dancing, swimming, jogging, running, and other leisure physical activities. We dichotomized leisure physical activity to having versus not having leisure physical activity. The mean and mode imputation method for continuous and categorical variables was used respectively, for missing covariates except for sex and age, because the corresponding missing data were less.

### Statistical analysis

Data are presented as the mean (standard deviation) and frequency (%) for continuous and categorical variables, respectively. Differences between groups were identified using the analysis of variance or the chi-square test.

We used linear and Poisson models with robust standard errors to examine the associations of the age at onset of overweight/obesity with BP and hypertension at the final survey, respectively, after adjusting for the length of follow-up, sex, and the characteristics reported in the final survey (age, Han ethnicity, urban residence, completion of upper middle school education and above, marital status, alcohol intake, smoking, household asset score, leisure physical activity, and fat intake) [14]. We estimated the standardized hypertension prevalence according to the groups classified by onset age, using the aforementioned covariate-adjusted Poisson model with a robust standard error. To assess the association between hypertension prevalence and the age at onset of overweight and obesity, we assigned values of 0, 1, 2 and 3 to the four groups (non-overweight/non-obesity, onset at ≥ 47 years, onset at 38–47 years and onset at < 38 years), respectively. Subsequently, we considered the age at onset of overweight and obesity as a continuous variable and used the covariate-adjusted Poisson model with a robust standard error to calculate the relative risk (RR) and 95% confidence interval (CI).

To test the robustness of our findings, we performed a sensitivity analysis. First, we excluded 206 participants who had antihypertensive medications on final survey and repeated all analyses. Second, we excluded 86 participants with new-onset obesity and repeated all analyses. Third, we assessed the combined effects of ages at onset of overweight and obesity and corresponding weight status on final survey on the incidence of hypertension. Fourth, we used waist circumference to define overweight and obesity (males: ≥85 cm; females: ≥80 cm) based on the Chinese criteria of weight for adults [15]. We calculated the tertiles of onset age at overweight and obesity defined by waist circumference. The upper and lower tertile cut-offs were 49 and 40 years, respectively. Participants were classified into four groups based on the age at onset of overweight/obesity. The four groups were those comprising the non-overweight/non-obese population and those with onset ages of < 40 years, 40–49 years and ≥ 49 years. Fifth, we examined the association between lifetime overweight/obesity exposure in terms of overweight/obese-years and the risk of hypertension. As described previously [16], overweight/obese-years was calculated as the defined degree of overweight/obesity [if the BMI < 24 kg/m^2^, the degree was zero; if the BMI ≥ 24 kg/m^2^, the degree was the BMI minus 23 kg/m^2^] multiplied by the defined duration of overweight/obesity. Sixth, we analyzed the association of weight gain, as defined by BMI difference between the final and first survey years, and the length of overweight/obesity exposure with incident hypertension [16]. Seventh, the severity of hypertension was classified as grade 1, 2, or 3 via the 2018 Chinese Guidelines for Prevention and Treatment of Hypertension [12]. Using the same guidelines, the pattern of hypertension was classified as isolated systolic hypertension, isolated diastolic hypertension, or combined systolic and diastolic hypertension [12]. We used the covariate-adjusted multinomial logit model to assess the association between the age at onset of overweight/obesity and the severity and pattern of hypertension after excluding individuals taking antihypertensive medications. Eighth, we conducted a sensitivity analysis stratified by sex.

We performed all analyses with SAS software version 9.4 (SAS Institute, Cary, NC, USA). A two-tailed *P* value of < 0.05 was considered statistically significant.

## Results

A total of 6,700 adults were included in the study (48.4% were male; mean age at the first survey, 36.9 years; mean SBP and DBP at the first survey, 109.8 and 71.6 mmHg, respectively; mean BMI at the first survey, 20.6 kg/m^2^). There were 2,284 new-onset overweight and obesity cases and 2,268 incident cases of hypertension during an average 13.8-year follow-up period among 6700 participants. The mean age at onset of overweight/obesity and hypertension was 43.6 and 54.9 years, respectively. The analysis cohort comprised 2,903 participants without new-onset overweight/obesity and hypertension, 1,529 participants with new-onset overweight/obesity and without new-onset hypertension, 1,513 participants without new-onset overweight/obesity and with new-onset hypertension, and 755 participants with new-onset overweight/obesity and hypertension (Figure S1).

The characteristics on final survey of the groups classified as overweight and obesity onset age are summarized in Table 1. In general, individuals with early-onset overweight/obesity (onset age of < 38 years) were more likely to be men and to have a higher BMI in the final survey.

**Table 1:** Characteristics on final survey stratified by onset age of overweight and obesity

	All participants	Onset age of overweight and obesity				
		No overweight and obesity	< 38 years	38–47 years	≥ 47 years	*P*
N	6700	4416	748	719	817	
Male participants (%)	48.4	48.9	51.3	46.5	44.3	0.022
Age (years)	50.7 (13.6)	51.0 (14.6)	39.5 (7.2)	48.9 (5.9)	61.3 (8.3)	< 0.001
Han Nationality (%)	85.9	85.2	89.4	87.2	85.3	0.013
Length of follow-up (years)	13.8 (6.5)	12.7 (6.5)	14.4 (5.7)	16.7 (5.7)	16.7 (5.9)	< 0.001
Urban residence (%)	29.9	29.3	31.4	29.2	32.6	0.208
Completed Upper middle school and above (%)	23.5	23.0	29.1	26.7	18.2	< 0.001
Marital status (%)						< 0.001
Never married	4.6	5.5	7.0	1.5	0.6	
Married	85.7	83.5	91.2	94.9	84.7	
Divorced/Separated/Widowed	9.6	11.0	1.9	3.6	14.7	
Household asset score	3.7 (1.7)	3.5 (1.8)	4.2 (1.5)	4.3 (1.5)	3.9 (1.6)	< 0.001
Drinking (%)	32.7	33.2	36.2	32.3	27.3	0.001
Smoking (%)						< 0.001
Never	67.5	65.8	66.6	73.4	72.6	
Past	2.5	2.5	2.1	1.8	3.7	
Current	30.0	31.7	31.3	24.8	23.8	
Leisure physical activity (%)	16.4	15.3	18.6	17.3	19.3	0.008
Fat intake (g/day)	70.9 (39.2)	69.9 (32.7)	72.8 (28.2)	73.4 (29.5)	72.7 (72.9)	< 0.001
BMI (kg/m^2^)	22.3 (3.1)	20.8 (1.9)	25.8 (3.2)	25.3 (3.0)	24.8 (2.6)	< 0.001
SBP (mm Hg)	123.7 (16.9)	122.9 (17.4)	122.0 (13.8)	124.4 (16.2)	129.0 (16.9)	< 0.001
DBP (mm Hg)	80.0 (10.7)	79.2 (10.9)	81.6 (9.9)	81.8 (10.3)	81.2 (10.1)	< 0.001
Hypertension (%)	33.9	34.3	28.7	31.3	38.6	< 0.001

BMI, body mass index; DBP, diastolic blood pressure; SBP, systolic blood pressure. Data were shown as means (SDs) and frequencies (%) for continuous and categorical variables, respectively. Differences were compared using the analysis of variance or chi-square test

**Table 2:** Association of overweight and obesity onset age with subsequent BP levels and hypertension

	No overweight and obesity	< 38 years	38–47 years	≥ 47 years
SBP				
β (SE)*	Ref	5.56 (0.64)	4.24 (0.64)	2.84 (0.62)
*P*		< 0.001	< 0.001	< 0.001
DBP				
β (SE)*	Ref	3.92 (0.43)	3.91 (0.43)	2.27 (0.41)
*P*		< 0.001	< 0.001	< 0.001
Hypertension				
Prevalence (%)*	26.6	38.6	35.9	30.9
RR (95% CI)*	Ref	1.45 (1.28, 1.65)	1.35 (1.21, 1.52)	1.16 (1.06, 1.28)
*P*		< 0.001	< 0.001	0.002
Trend in hypertension **				
RR (95% CI) *	1.14 (1.10, 1.18)			
*P* for trend	< 0.001			

BP, blood pressure; CI, confidence interval; DBP, diastolic blood pressure; RR, risk ratio; SBP, systolic blood pressure; β, unstandardized regression coefficients. * Adjusted for the length of follow-up, sex, and age, drinking, smoking, Han nationality, urban residence, completed upper middle school and above, marital status, household asset score, leisure physical activity and fat intake on final survey. ** We assigned values of 0, 1, 2 and 3 to the four groups (non-overweight/non-obesity, onset at ≥ 47 years, onset at 38–47 years and onset at < 38 years), respectively. Subsequently, we considered the age at onset of overweight and obesity as a continuous variable and used the covariate-adjusted Poisson model with a robust standard error to assess the trend in prevalence

The association between the age at onset of overweight/obesity and BP or hypertension is shown in Table 2. The participants with an age at onset of overweight/obesity of < 38 years, 38–47 years and ≥ 47 years had a higher SBP and DBP than those without overweight/obesity (*P*s < 0.001). The standardized hypertension prevalence was 38.6%, 35.9%, 30.9% and 26.6% in participants with an overweight/obesity onset age of < 38 years, 38–47 years and ≥ 47 years and without overweight/obesity, respectively. Individuals with an age at onset of overweight/obesity of < 38 years, 38–47 years and ≥ 47 years had a RR (95% CI) of 1.45 (1.28–1.65), 1.35 (1.21–1.52) and 1.16 (1.06–1.28), respectively, for hypertension compared with those without overweight/obesity. The risk of hypertension increased linearly with a decrease in the age at onset of overweight/obesity (*P* < 0.001 for trend).

We excluded individuals taking antihypertensive medications and obtained similar results (Table S1). We also conducted sensitivity analyses after excluding the participants with new-onset obesity. The results did not change substantially (Table S2). Additionally, to assess the effects of weight change on the incidence of hypertension, we excluded 424 individuals with new-onset overweight and obesity on the final survey because the weight change data after new-onset overweight and obesity were unavailable for these participants. 75.5%, 71.2% and 62.9% of the participants with an age at onset of overweight/obesity of < 38 years, 38–47 years and ≥ 47 years remained to have overweight/obesity on final survey respectively (Table S3). The difference in the aforementioned prevalence was significant (*P* < 0.001). Compared with the participants with persistent normal weight, those with early-onset overweight/obesity (onset age < 38 years) as well as overweight/obesity on the final survey had a higher risk of hypertension; those who had early-onset overweight/obesity and returned to normalcy on the final survey had no higher risk (Table S3). Moreover, the sensitivity analyses results were similar to the main findings when using waist circumference to define overweight and obesity (Table 2 and S4). The mean overweight/obese-years were 0, 14.8, 10.6, 7.0 (kg/m^2^×year) for the non-overweight/non-obese individuals and those with overweight/obesity onset ages of < 38 years, 38–47 years and ≥ 47 years, respectively (*P* < 0.001). We also found that overweight/obese-years was positively related to the risk of hypertension (Table S5). The weight gain or length of overweight/obesity exposure was significantly associated with incident hypertension (Table S6). The length of exposure was also significantly correlated with incident hypertension after adjusting for weight gain (Table S6). The mean length of overweight/obesity exposure was 0, 7.17, 6.19, 5.34 (years) for the non-overweight/non-obese individuals and those with overweight/obesity onset ages of < 38 years, 38–47 years, and ≥ 47 years, respectively (*P* < 0.001). Generally, we also observed that participants with early-onset overweight/obesity were more likely to have hypertension, regardless of the hypertension pattern or severity (Table S7 and S8). The sensitivity analyses results stratified by sex did not change significantly (Table S9).

## Discussion

In this study, we determined that participants who were classified as overweight or obese with an onset age of < 38 years had a higher risk of hypertension relative to those who were not classified as overweight or obese. We also identified an increasing trend in future hypertension risk with a decrease in age at onset of overweight/obesity.

Our results are in line with those from a 1946 British birth cohort study, which found that participants who were described as overweight with early onset had a higher mean BP later in life than those who were described as late-onset overweight [17]. Similarly, a 1958 British birth cohort study further showed that a high BMI and a high BMI gain at any life stage were related to an increase in BP in adulthood [18]. A previous study used data from patients with obesity who attended a weight management clinic to show that the onset of excess weight at a young age contributed to later extreme obesity and a high SBP [19]. The Johns Hopkins Precursors Study showed that a higher BMI at the age of 25 years was associated with an increased risk of future hypertension [20]. Our findings were partly supported by previous studies, which have indicated that early-onset obesity is associated with a higher risk of type 2 diabetes, metabolic syndrome and major chronic diseases than late-onset obesity [21–24]. Based on the aforementioned evidence, early-onset obesity is considered as a public health challenge because it introduces hypertension and diabetes mellitus at a premature stage in life [25].

Recently, Kailuan cohort study reported that early age at onset of overweight was related to higher risk of hypertension, which was consistent with our study [9]. However, our study provided additional information. First, previous studies have only used BMI to assess the weight status [9, 17–20]; however, BMI is not a perfect measure of abdominal fat. The advantage of this study is the use of BMI and waist circumference to evaluate the age at onset of general and abdominal overweight/obesity, respectively. The corresponding sensitivity analysis results were similar to our main analysis findings (Table 2 and S4). Second, our study assessed the association of the age at onset of overweight/obesity and the corresponding weight status in the final survey with the incidence of hypertension. Our results demonstrated that individuals with early-onset overweight/obesity tended to maintain overweight/obesity, and individuals with persistent overweight/obesity had a higher risk of hypertension than those with the persistent normal weight (Table S3). However, compared with the participants with persistent normal weight, those who had early-onset overweight/obesity (onset age < 38 years) and returned to normalcy on the final survey, had no higher risk of hypertension (Table S3). Our findings underscored the importance of loss weight in the prevention of hypertension among participants with early-onset overweight/obesity. Third, this study determined that individuals with early-onset overweight/obesity had more lifetime overweight/obesity exposure in terms of overweight/obese-years, which partly explains their higher risk of hypertension in comparison with individuals with late-onset overweight/obesity (Table S5). Fourth, our study also revealed that the length of overweight/obesity exposure is more detrimental to incident hypertension given the limited extent of weight gain (mean and median: 2.69 and 2.18 kg/m^2^, respectively, for 4,986 individuals with increasing weight gain; Table S6), which partly explains our main findings given the longer duration of overweight/obesity exposure in participants with early-onset overweight/obesity. Fifth, we confirmed that participants with early-onset overweight/obesity were more likely to have hypertension, regardless of the pattern or severity (Table S7 and S8). Sixth, the sensitivity analyses results stratified by sex did not change significantly (Table S9).

Several potential mechanisms might explain our findings. Early-onset overweight and obesity has a high cumulative effect or a longer duration of overweight and obesity, because individuals classified as overweight and obese tend to maintain their weight status [5, 6]. Previous studies have shown that an increase in the duration of overweight and obesity is associated with a higher risk of adverse health outcomes [5, 26]. Our study provides evidence to support the above-mentioned assertion (Table S5 and S6). Additionally, a previous study has shown that a stronger genetic component predisposes to early-onset overweight and obesity [27]. Obesity and hypertension share common susceptibility genes, such as genes Jun proto-oncogene (JUN), and serine/threonine kinase 1 (AKT1) [28]. Moreover, overweight and obesity onset at different stages of life are associated with various types of health risk [5, 29]. Sensitive-period models have shown that exposures exerted the greatest effects during rapid development in humans [29]. Taken together, participants with early-onset overweight/obesity were more likely to possess genes associated with cardiometabolic risk and have damage for long-term and critical period exposure, causing adverse metabolic profile, in comparison with participants with late-onset overweight/obesity.

Our study has several strengths, including its prospective cohort study design and rigorous data collection process. However, some limitations in this study should also be recognized. First, the age at onset of overweight/obesity was assessed by objectively measuring weight and height during the survey waves. However, overweight/obesity onset occurring prior to the survey wave may have affected our results. Second, some potential confounding variables (e.g. salt intake) were not adjusted. One noteworthy observation in our study was that the prevalence of hypertension was lower in participants with onset ages of < 38 years compared to those with the other three groups (Table 1), which might be affected by covariates. For example, the prevalence of hypertension increases with age [30]. Participants with onset ages of < 38 years were youngest than those with the other three groups (Table 1). Although we adjusted for some covariates (e.g. age), other potential confounding variables (e.g. salt intake) might affect our findings. Third, there is no widely accepted definition of early-onset overweight/obesity. Consequently, we used the upper and lower tertiles as relevant cut-offs. Future studies are needed to address this issue. Finally, the generalizability of our findings should be verified with caution, given that our study was carried out in the Chinese population. Future studies in other populations are needed to confirm our findings.

## Conclusions

In summary, we determined that participants who were classified as overweight or obese with an onset age of < 38 years, 38–47 years, and ≥ 47 years had a higher risk of hypertension relative to those who were not classified as overweight or obese. We also identified an increasing trend in future hypertension risk with a decrease in age at onset of overweight/obesity. Our findings emphasize the importance of assessing the age at onset of overweight and obesity to prevent hypertension.

## Electronic supplementary material

Below is the link to the electronic supplementary material.

Additional file 1: supplement Figure S1, and Table S1-S9

## Data Availability

The datasets generated and/or analyzed during the current study are publicly available in the https://www.cpc.unc.edu/projects/china/data/datasets/data-downloads-registration.

## Abbreviations

BMI: body mass index
CHNS: China Health and Nutrition Survey
SBP: systolic blood pressure
DBP: diastolic blood pressure
