# Supplementary material for: Association between the age at onset of overweight and obesity and the subsequent risk of hypertension in Chinese adults
Source: BMC Cardiovasc Disord. 2023 Jun 30;23:333. doi: 10.1186/s12872-023-03347-z (PMC10311763; doi:10.1186/s12872-023-03347-z)
Supplement: Supplementary file 1 — Additional file 1: supplement Figure S1, and Table S1-S9 [file 12872_2023_3347_MOESM1_ESM.docx]

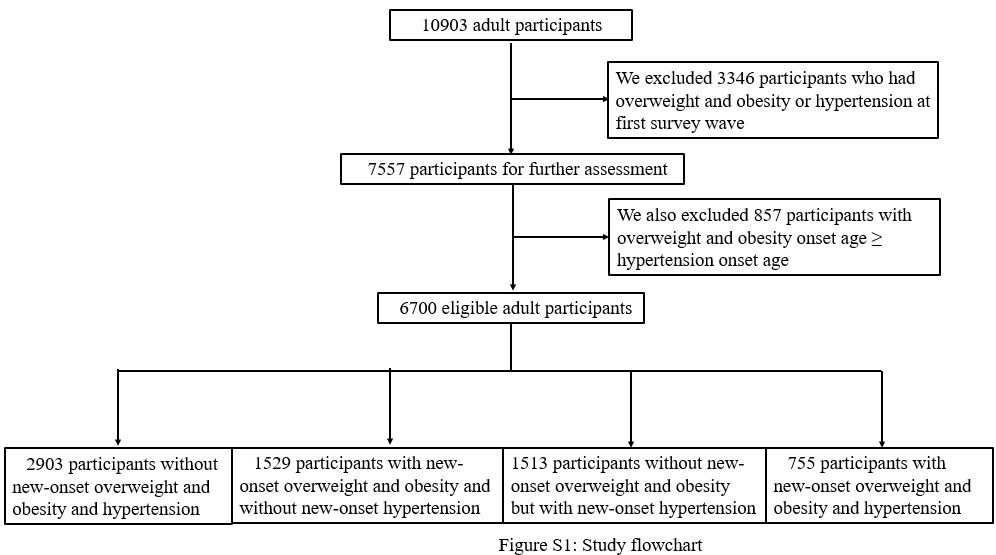


| Table S1. Association of overweight and obesity onset age with subsequent BP levels and hypertension after excluding individuals with taking antihypertensive medications on final survey | | | | |  |
| --- | --- | --- | --- | --- | --- |
|  | No overweight and obesity | < 38 years | 38-47 years | ≥47 years |  |
| N | 4300 | 732 | 690 | 772 |  |
| SBP |  |  |  |  |  |
| β (SE)* | Ref | 5.41 (0.63) | 3.85 (0.64) | 2.77 (0.62) |  |
| *P* |  | <0.001 | <0.001 | <0.001 |  |
| DBP |  |  |  |  |  |
| β (SE)* | Ref | 3.82 (0.43) | 3.65 (0.43) | 2.21 (0.41) |  |
| *P* |  | <0.001 | <0.001 | <0.001 |  |
| Hypertension |  |  |  |  |  |
| Prevalence (%)* | 24.4 | 35.8 | 32.5 | 27.9 |  |
| RR (95% CI)* | Ref | 1.46 (1.28, 1.67) | 1.33 (1.18, 1.51) | 1.14 (1.03, 1.26) |  |
| *P* |  | <0.001 | <0.001 | 0.014 |  |
| Trend in hypertension ** |  |  |  |  |  |
| RR (95% CI) * | 1.14 (1.10, 1.18) | | | |  |
| *P* for trend | <0.001 | | | |  |
| BP, blood pressure; CI, confidence interval; DBP, diastolic blood pressure; RR, risk ratio; SBP, systolic blood pressure; β, unstandardized regression coefficients. * Adjusted for the length of follow‐up, sex, and age, drinking, smoking, Han nationality, urban residence, completed upper middle school and above, marital status, household asset score, leisure physical activity and fat intake on final survey. ** We assigned values of 0, 1, 2 and 3 to the four groups (non-overweight/non-obesity, onset at ≥47 years, onset at 38–47 years and onset at <38 years), respectively. Subsequently, we considered the age at onset of overweight and obesity as a continuous variable and used the covariate-adjusted Poisson model with a robust standard error to assess the trend in prevalence. | | | | |  |
|  |  |  |  |  |  |
|  |  |  |  |  |  |
|  |  |  |  |  |  |
|  |  |  |  |  |  |

| Table S2. Association of overweight onset age with subsequent BP levels and hypertension after excluding individuals with new-onset obesity | | | | |
| --- | --- | --- | --- | --- |
|  | No overweight and obesity | < 38 years | 38-47 years | ≥47 years |
| N | 4416 | 717 | 693 | 788 |
| SBP |  |  |  |  |
| β (SE)* | Ref | 5.62 (0.65) | 4.30 (0.65) | 2.97 (0.62) |
| *P* |  | <0.001 | <0.001 | <0.001 |
| DBP |  |  |  |  |
| β (SE)* | Ref | 3.88 (0.44) | 3.91 (0.43) | 2.29 (0.42) |
| *P* |  | <0.001 | <0.001 | <0.001 |
| Hypertension |  |  |  |  |
| Prevalence (%)* | 26.6 | 38.8 | 36.3 | 31.2 |
| RR (95% CI)* | Ref | 1.46 (1.28, 1.66) | 1.36 (1.21, 1.53) | 1.17 (1.07, 1.29) |
| *P* |  | <0.001 | <0.001 | 0.001 |
| Trend in hypertension ** |  |  |  |  |
| RR (95% CI) * | 1.15 (1.10, 1.19) | | | |
| *P* for trend | <0.001 | | | |
| BP, blood pressure; CI, confidence interval; DBP, diastolic blood pressure; RR, risk ratio; SBP, systolic blood pressure; β, unstandardized regression coefficients. * Adjusted for the length of follow‐up, sex, and age, drinking, smoking, Han nationality, urban residence, completed upper middle school and above, marital status, household asset score, leisure physical activity and fat intake on final survey. ** We assigned values of 0, 1, 2 and 3 to the four groups (non-overweight/non-obesity, onset at ≥47 years, onset at 38–47 years and onset at <38 years), respectively. Subsequently, we considered the age at onset of overweight and obesity as a continuous variable and used the covariate-adjusted Poisson model with a robust standard error to assess the trend in prevalence. | | | | |
|  |  |  |  |  |
|  |  |  |  |  |
|  |  |  |  |  |
|  |  |  |  |  |

| Table S3. Association of overweight and obesity onset age and corresponding weight status on final survey with hypertension after excluding individuals with new-onset overweight/obesity on final survey | | | | | |
| --- | --- | --- | --- | --- | --- |
| Onset age | Weight status on final survey | N | Prevalence (%)* | RR (95% CI) * | *P* |
| No overweight and obesity | No overweight and obesity | 4416 | 27.3 | Ref |  |
| < 38 years | No overweight and obesity | 158 | 32.4 | 1.19 (0.91, 1.56) | 0.212 |
| < 38 years | Overweight and obesity | 488 | 48.3 | 1.77 (1.56, 2.02) | <0.001 |
| 38-47 years | No overweight and obesity | 168 | 33.0 | 1.21 (0.96, 1.52) | 0.102 |
| 38-47 years | Overweight and obesity | 416 | 48.2 | 1.77 (1.57, 1.99) | <0.001 |
| ≥47 years | No overweight and obesity | 234 | 35.7 | 1.31 (1.13, 1.51) | <0.001 |
| ≥47 years | Overweight and obesity | 396 | 42.9 | 1.57 (1.42, 1.74) | <0.001 |
| CI, confidence interval; RR, risk ratio. * Adjusted for the length of follow‐up, sex, and age, drinking, smoking, Han nationality, urban residence, completed upper middle school and above, marital status, household asset score, leisure physical activity and fat intake on final survey. | | | | | |

| Table S4. Association of onset age of overweight and obesity defined by waist circumference with subsequent BP levels and hypertension | | | | |  |
| --- | --- | --- | --- | --- | --- |
|  | No overweight and obesity | < 40 years*** | 40-49 years*** | ≥49 years*** |  |
| N | 2459 | 982 | 960 | 1020 |  |
| SBP |  |  |  |  |  |
| β (SE)* | Ref | 4.71 (0.61) | 3.03 (0.59) | 2.79 (0.63) |  |
| *P* |  | <0.001 | <0.001 | <0.001 |  |
| DBP |  |  |  |  |  |
| β (SE)* | Ref | 3.11 (0.41) | 3.39 (0.40) | 1.74 (0.42) |  |
| *P* |  | <0.001 | <0.001 | <0.001 |  |
| Hypertension |  |  |  |  |  |
| Prevalence (%)* | 20.6 | 31.1 | 29.6 | 24.8 |  |
| RR (95% CI)* | Ref | 1.51 (1.31, 1.74) | 1.44 (1.27, 1.63) | 1.21 (1.09, 1.34) |  |
| *P* |  | <0.001 | <0.001 | <0.001 |  |
| Trend in hypertension ** |  |  |  |  |  |
| RR (95% CI) * | 1.16 (1.12, 1.21) | | | |  |
| *P* for trend | <0.001 | | | |  |
| BP, blood pressure; CI, confidence interval; DBP, diastolic blood pressure; RR, risk ratio; SBP, systolic blood pressure; β, unstandardized regression coefficients. * Adjusted for the length of follow‐up, sex, and age, drinking, smoking, Han nationality, urban residence, completed upper middle school and above, marital status, household asset score, leisure physical activity and fat intake on final survey. ** We assigned values of 0, 1, 2 and 3 to the four groups (non-overweight/non-obesity, onset at ≥49 years, onset at 40–49 years and onset at <40 years), respectively. Subsequently, we considered the age at onset of overweight and obesity as a continuous variable and used the covariate-adjusted Poisson model with a robust standard error to assess the trend in prevalence. *** We calculated the tertiles of onset age. The upper and lower tertile cut-offs were 49 and 40 years, respectively. | | | | |  |
|  |  |  |  |  |  |
|  |  |  |  |  |  |
|  |  |  |  |  |  |
|  |  |  |  |  |  |

| Table S5. Association between lifetime overweight/obesity exposure in terms of overweight/obese-years and the risk of hypertension | | | | |  |
| --- | --- | --- | --- | --- | --- |
|  | N | RR (95% CI)* | *P* | |  |
| Continuous overweight/obese-years (kg/m^2^×year) | 6700 | 1.017 (1.014, 1.020) | <0.001 | |  |
| Overweight/obese-years (kg/m^2^×year) |  |  | |  |  |
| 0 | 5389 | Ref | |  |  |
| <12.97** | 655 | 1.580 (1.439, 1.735) | | <0.001 |  |
| ≥12.97 | 656 | 1.968 (1.779, 2.178) | | <0.001 |  |
| Trend*** | 6700 | 1.429 (1.364, 1.498) | | <0.001 |  |
| CI, confidence interval; RR, risk ratio; * Adjusted for the length of follow‐up, sex, and age, drinking, smoking, Han nationality, urban residence, completed upper middle school and above, marital status, household asset score, leisure physical activity and fat intake on final survey. ** 12.97 was median after excluding the individual whose overweight/obese-years was equal to zero. *** We assigned values of 0, 1, and 2 to the three groups (0, overweight/obese-years <12.97 and overweight/obese-years ≥12.97), respectively. Subsequently, we considered the aforementioned variable as a continuous variable and used the covariate-adjusted Poisson model with a robust standard error to assess the trend. | | | | |  |
|  |  |  |  |  |  |
|  |  |  |  |  |  |
|  |  |  |  |  |  |
|  |  |  |  |  |  |

| Table S6. Association of the weight gain and length of overweight/obesity exposure with the risk of hypertension | | | | |
| --- | --- | --- | --- | --- |
|  | N | RR (95% CI)* | *P* | |
| Model 1: weight gain (kg/m^2^) |  |  |  | |
| 0 or <0 | 1714 | Ref |  | |
| <2.18** | 2490 | 1.07 (0.99, 1.15) | 0.095 | |
| ≥2.18 | 2496 | 1.24 (1.14, 1.35) | <0.001 | |
| Model 2: length of overweight/obesity exposure (years) |  |  |  | |
| 0 | 5389 | Ref | |  |
| <6*** | 613 | 1.66 (1.51, 1.82) | | <0.001 |
| ≥6 | 698 | 1.84 (1.67, 2.04) | | <0.001 |
| Model 3 |  |  | |  |
| weight gain (kg/m^2^) |  |  | |  |
| 0 or <0 | 1714 | Ref | |  |
| <2.18** | 2490 | 1.02 (0.95, 1.10) | | 0.564 |
| ≥2.18 | 2496 | 0.98 (0.89, 1.08) | | 0.688 |
| length of overweight/obesity exposure (years) |  |  | |  |
| 0 | 5389 | Ref | |  |
| <6*** | 613 | 1.68 (1.52, 1.85) | | <0.001 |
| ≥6 | 698 | 1.87 (1.68, 2.09) | | <0.001 |
| CI, confidence interval; RR, risk ratio; Wain gain was calculating as BMI difference between the final and first survey years. *Adjusted for the length of follow‐up, sex, and age, drinking, smoking, Han nationality, urban residence, completed upper middle school and above, marital status, household asset score, leisure physical activity and fat intake on final survey. ** 2.18 kg/m^2^ was median after excluding the individual whose weight gain was ≤ 0. *** 6 years was median after excluding the individual whose length of overweight/obesity exposure was equal to 0. | | | | |
|  |  |  |  |  |
|  |  |  |  |  |
|  |  |  |  |  |
|  |  |  |  |  |
| Table S7. Association of overweight and obesity onset age with the severity of hypertension after excluding individuals with taking antihypertensive medications on final survey | | | | |
|  | N | OR (95% CI)* | *P* | |
| Normotension (N=4432) vs grade 1 hypertension (N=1721) |  |  |  | |
| Overweight and obesity onset age |  |  |  | |
| No overweight and obesity | 4095 | Ref |  | |
| < 38 years | 697 | 1.76 (1.43, 2.17) | <0.001 | |
| 38-47 years | 649 | 1.44 (1.17, 1.78) | <0.001 | |
| ≥47 years | 712 | 1.07 (0.88, 1.30) | 0.515 | |
| Trend** |  | 1.20 (1.12, 1.27) | <0.001 | |
| Normotension (N=4432) vs grade 2 and 3 hypertension (N=341) |  |  | |  |
| Overweight and obesity onset age |  |  | |  |
| No overweight and obesity | 3108 | Ref | |  |
| < 38 years | 568 | 2.76 (1.82, 4.20) | | <0.001 |
| 38-47 years | 535 | 2.34 (1.60, 3.42) | | <0.001 |
| ≥47 years | 562 | 1.60 (1.16, 2.22) | | 0.005 |
| Trend** |  | 1.45 (1.28, 1.64) | | <0.001 |
| CI, confidence interval; OR, odds ratio; grade 1 hypertension, 160 mm Hg >systolic blood pressure ≥ 140 mm Hg and/or 100 mm Hg >diastolic blood pressure≥ 90 mm Hg; grade 2 and 3 hypertension, systolic blood pressure ≥ 160 mm Hg and/or diastolic blood pressure ≥ 100 mm Hg. We considered grade 2 and 3 hypertension as one category due to small sample of grade 3 hypertension (n=70). *Adjusted for the length of follow‐up, sex, and age, drinking, smoking, Han nationality, urban residence, completed upper middle school and above, marital status, household asset score, leisure physical activity and fat intake on final survey. ** We assigned values of 0, 1, 2 and 3 to the four groups (non-overweight/non-obesity, onset at ≥47 years, onset at 38–47 years and onset at <38 years), respectively. | | | | |
|  |  |  |  |  |
|  |  |  |  |  |
|  |  |  |  |  |
|  |  |  |  |  |

| Table S8. Association of overweight and obesity onset age with the pattern of hypertension after excluding individuals with taking antihypertensive medications on final survey | | | | |
| --- | --- | --- | --- | --- |
|  | N | OR (95% CI)* | | *P* |
| Normotension (N=4432) vs isolated systolic hypertension (N=632) |  |  | |  |
| Overweight and obesity onset age |  |  | |  |
| No overweight and obesity | 3352 | Ref | |  |
| < 38 years | 557 | 1.29 (0.82, 2.03) | | 0.273 |
| 38-47 years | 542 | 1.54 (1.10, 2.16) | | 0.013 |
| ≥47 years | 613 | 1.19 (0.93, 1.53) | | 0.176 |
| Trend** |  | 1.16 (1.03, 1.30) | | 0.013 |
| Normotension (N=4432) vs isolated diastolic hypertension (N=877) |  |  |  | |
| Overweight and obesity onset age |  |  |  | |
| No overweight and obesity | 3499 | Ref |  | |
| < 38 years | 653 | 1.88 (1.47, 2.40) | <0.001 | |
| 38-47 years | 579 | 1.51 (1.15, 1.97) | 0.003 | |
| ≥47 years | 578 | 1.07 (0.81, 1.41) | 0.653 | |
| Trend** |  | 1.23 (1.14, 1.32) | <0.001 | |
| Normotension (N=4432) vs combined systolic and diastolic hypertension (N=553) |  |  |  | |
| Overweight and obesity onset age |  |  |  | |
| No overweight and obesity | 3255 | Ref |  | |
| < 38 years | 588 | 2.26 (1.62, 3.17) | <0.001 | |
| 38-47 years | 557 | 1.95 (1.43, 2.65) | <0.001 | |
| ≥47 years | 585 | 1.25 (0.95, 1.65) | 0.118 | |
| Trend** |  | 1.33 (1.21, 1.47) | <0.001 | |
| CI, confidence interval; OR, odds ratio; isolated systolic hypertension, systolic blood pressure ≥ 140 mm Hg and diastolic blood pressure< 90 mm Hg; isolated diastolic hypertension, systolic blood pressure <140 mm Hg and diastolic blood pressure ≥90 mm Hg; combined systolic and diastolic hypertension, systolic blood pressure ≥140 mm Hg and diastolic blood pressure ≥90 mm Hg.  *Adjusted for the length of follow‐up, sex, and age, drinking, smoking, Han nationality, urban residence, completed upper middle school and above, marital status, household asset score, leisure physical activity and fat intake on final survey.  ** We assigned values of 0, 1, 2 and 3 to the four groups (non-overweight/non-obesity, onset at ≥47 years, onset at 38–47 years and onset at <38 years), respectively. | | | | |
|  |  |  |  |  |
|  |  |  |  |  |
|  |  |  |  |  |
|  |  |  |  |  |

| Table S9. Association of overweight and obesity onset age with hypertension stratified by sex | | | | |
| --- | --- | --- | --- | --- |
|  | No overweight and obesity | < 38 years | 38-47 years | ≥47 years |
| Male |  |  |  |  |
| N | 2161 | 384 | 334 | 362 |
| Hypertension |  |  |  |  |
| RR (95% CI)* | Ref | 1.42 (1.20, 1.68) | 1.26 (1.08, 1.48) | 1.10 (0.96, 1.26) |
| *P* |  | <0.001 | 0.004 | 0.188 |
| Trend** |  |  |  |  |
| RR (95% CI) * | 1.12 (1.07, 1.18) | | | |
| *P* for trend | <0.001 | | | |
| Female |  |  |  |  |
| N | 2255 | 364 | 385 | 455 |
| Hypertension |  |  |  |  |
| RR (95% CI)* | Ref | 1.41 (1.15, 1.72) | 1.42 (1.20, 1.68) | 1.20 (1.05, 1.36) |
| *P* |  | <0.001 | <0.001 | 0.007 |
| Trend** |  |  |  |  |
| RR (95% CI) * | 1.15 (1.09, 1.21) | | | |
| *P* for trend | <0.001 | | | |
| CI, confidence interval; RR, risk ratio. * Adjusted for the length of follow‐up, and age, drinking, smoking, Han nationality, urban residence, completed upper middle school and above, marital status, household asset score, leisure physical activity and fat intake on final survey. ** We assigned values of 0, 1, 2 and 3 to the four groups (non-overweight/non-obesity, onset at ≥47 years, onset at 38–47 years and onset at <38 years), respectively. Subsequently, we considered the age at onset of overweight and obesity as a continuous variable and used the covariate-adjusted Poisson model with a robust standard error to assess the trend in prevalence. | | | | |
|  |  |  |  |  |
|  |  |  |  |  |
|  |  |  |  |  |
|  |  |  |  |  |
